# Genetic diagnosis and clinical evaluation of severe fetal akinesia syndrome

**DOI:** 10.1002/pd.5809

**Published:** 2020-09-10

**Authors:** Theresa Reischer, Sandra Liebmann‐Reindl, Dieter Bettelheim, Sukirthini Balendran‐Braun, Berthold Streubel

**Affiliations:** ^1^ Department of Obstetrics and Feto‐Maternal Medicine Medical University of Vienna Vienna Austria; ^2^ Core Facility Genomics Medical University of Vienna Vienna Austria; ^3^ Clinical Institute of Pathology Medical University of Vienna Vienna Austria

## Abstract

**Objective:**

In this retrospective study, we describe the clinical course, ultrasound findings and genetic investigations of fetuses affected by fetal akinesia.

**Materials and Methods:**

We enrolled 22 eukaryotic fetuses of 18 families, diagnosed with fetal akinesia between 2008 and 2016 at the Department of Obstetrics and Feto‐Maternal Medicine at the Medical University of Vienna. Routine genetic evaluation included karyotyping and chromosomal microarray analysis. Retrospectively, exome sequencing was performed in the index case of 11 families, if stored DNA was available. Confirmation analyses and genetic diagnosis of siblings were performed by using Sanger sequencing.

**Results:**

Whole exome sequencing identified pathogenic variants of *CNTN1*, *RYR1*, *NEB*, *GLDN*, *HRAS and TNNT3* in six cases of 11 families. In three of these families, the variants were confirmed in the respective sibling.

**Conclusions:**

The present study demonstrates a high diagnostic yield of exome sequencing in fetuses affected by akinesia syndrome, especially if family history is positive. Still, in a large part the underlying genetic cause remained unknown, whereas precise clinical evaluation in combination with exome sequencing shows to be the best tool to find the disease causing variants.

1

**What's already known about this topic?**

- The diagnostic yield of prenatally used whole exome sequencing varies tremendously between different studies.

**What does this study add?**

- We demonstrate a high diagnostic yield using exome sequencing, in fetuses affected by severe akinesia syndrome. In eukaryote cases with the suspicion of fetal akinesia syndrome, especially if family history is positive, whole exome sequencing should be considered as next diagnostic step. The chance of a positive result within this indication should be discussed during pretest genetic counseling.

## 2 INTRODUCTION

The term fetal akinesia represents a category of disorders within a broad spectrum of diseases leading to reduced or absent fetal movements. This clinical finding is often recognized as a sequence of deformational changes related to reduced fetal movements called fetal akinesia deformations sequence (FADS). Many different alternative nomenclatures have been described in the literature including multiple congenital contractures (MCC), and most commonly arthrogryposis multiplex congenita (AMC). For the definition of AMC or FADS at least two or more joint contractures in different body areas have to be present.^1^, ^2^

The phenotype of FADS includes features like intrauterine growth restriction (IUGR), craniofacial anomalies, limb contractures, pulmonary hypoplasia, short umbilical cord, together with pregnancy complications such as polyhydramnios and abnormal intrauterine positioning. This phenotype can occur isolated or associated with additional organ system anomalies.^3^, ^4^, ^5^

Regarding prenatal diagnosis ultrasound findings of arthrogryposis, defined as multiple congenital contractures, club feet and clenched hands may be signs of decreased fetal movements. Moreover, nuchal edema, fetal hydrops and abnormal facial profile are observed in affected fetuses (FADS).^6^ Later in pregnancy maternal perception of decreased intrauterine movements and developing polyhydramnios are important indications.^7^ Previous investigations showed that prenatal MRI can visualize fetal akinesia and helps to identify associated findings, in particularly associated CNS abnormalities and lung hypolasia.^8^

Concerning the etiology of this heterogeneous presenting phenotype a variety of genetic, non‐genetic, parental and environmental causes are known. Environmental causes include circulating maternal antibodies to neurotransmitters, myelin and muscle proteins, restricted intrauterine space, drug use, maternal illness and ischemia.^9^ Regarding genetic etiologies generally FADS can result from defects at any point along the motor system.^10^ Although many well‐defined genetic syndromes, are associated with decreased or absent fetal movements, the genetic diagnostic rate of these cases remains low.^3^, ^6^, ^9^, ^10^ This is due to the rarity and the genetic heterogeneity of these phenotypes and compounded by the difficulty of assigning unspecific prenatal clinical findings to a defined syndrome^6^. In the last years, through the availability of next generation sequencing methods, continuously genes associated with neuromuscular disease have been identified.^11^

In this study, we comprehensively investigated 22 cases of prenatally diagnosed FADS and identified diverse genetic disorders responsible for this severe phenotype.

## 3 METHODS

Ethical approval was obtained from the Ethics Committee of the Medical University of Vienna (1496/2015).

### 3.1 Patient selection

This retrospective study included 22 cases of suspected FADS diagnosed at the Department of Gynecology and Obstetrics of the Medical University of Vienna between 2008 and 2016. Inclusion criteria were decreased or missing fetal movement initially revealed by ultrasound plus arthrogryposis of the limbs. MRI helped to confirm ultrasound findings and to identify associated anomalies. Fetuses without prenatal or postnatal genetic investigation or fetuses with an abnormal karyotype were excluded. Isolated club feet and isolated anomalies of the limbs were not included. For clinical evaluation ultrasound reports, stored images, autopsy reports, MRI reports and pediatric charts were used, if available.

### 3.2 Clinical routine genetic diagnosis

In all 22 affected fetuses, a genetic investigation was initiated through invasive prenatal diagnosis or postnatal sampling (amniotic fluid, chorionic villi, muscle tissue, blood). The first step of clinical routine genetic diagnosis included conventional karyotyping, using standard giemsa staining. At the second step, chromosomal microarray analysis was conducted in all of these cases within the scope of clinical routine diagnostics.

Of all 21 eukaryote fetuses with normal chromosomal microarray results, in 15 cases of 11 families stored genomic DNA samples were available and therefore whole exome sequencing (WES) was performed in the index case of each family. Confirmation analyses and genetic diagnosis of relatives like siblings were performed by using Sanger sequencing.^12^

Genomic DNA was isolated after clinical routine sampling from amniotic fluid or chorionic villi (prenatal), blood (postnatal) or muscle tissue (postmortem) using standard Qiagen DNA Isolation kits. DNA quantification was conducted using Qubit high‐sensitivity (HS)—fluorometric measurements.

Subsequently genomic DNA samples were subjected to WES using Illumina Nextera Rapid Capture Exome Kit for library preparation. Sequencing was performed with HiSeq 3000 platform using 100 bp paired‐end (PE) chemistry. Next‐Generation Sequencing data analysis was conducted using BWA Enrichment pipeline via Basespace Sequence Hub (Version: 2.1.1).^13^ For all datasets, only quality‐filter passed variants were considered for further analysis exclusively.

During variant evaluation, the focus was primarily on variants in genes known to cause fetal akinesia syndrome according to literature research, mainly via PubMed and OMIM database, which resulted in 43 genes reported to cause fetal akinesia syndrome at the time of analysis (Table S1). Additionally, variants in genes associated with congenital myopathy, muscle dystrophy and disease‐causing genes, associated with any kind of muscle involvement were screened for pathological variants. Furthermore, (especially if negative) after the first two steps of filtering, all data were analyzed for known pathogenic variants according to ClinVar and HGMD Databases. All other variants were classified according to ACMG guidelines.^14^

## 4 RESULTS

### 4.1 Clinical findings

In total 22 cases of suspected fetal akinesia syndrome in 18 families were included in this study. The clinical suspicion of FADS was established between 12 and 34 weeks of pregnancy (WoP), mainly depending on the time of referral to our department. The mean gestational age of clinical diagnosis was 23 WoP. Family history, in particular of FADS in a previous pregnancy was positive in eight of 18 families (44%). In two families, there were more than two affected fetuses. Overall consanguinity was known in four of 18 families (22%).

Polyhydramnios was the most common finding besides decreased or missing fetal movement, which was mandatory for the inclusion in this study, present in 17 of 22 cases (77%).

Concerning in utero growth, expected fetal weight was normal in 11 of 22 cases (50%), intrauterine growth restriction (IUGR; <5th Percentile) was present in two cases (9%), three fetuses were small for gestational age (SGA; <10th Percentile; 14%) and six fetuses had an expected fetal weight above the 90th percentile, defined as large for gestational age (respective 27%). The stomach, as an indicator of fetal ability of swallowing, showed absent or minimal filling in 14% (3/22). Abnormal fetal swallowing and increased amniotic fluid were associated in all of the three cases.

Increased nuchal translucency at first trimester screening was present in 45% (10/22). Eight of 22 fetuses (36%) developed signs of fetal hydrops such as skin edema, hydrothorax, ascites, pericardial or pleural effusion. Reduced lung volume or lung hypoplasia was present in 15 of 22 fetuses (68%). Summarized ultrasound findings and details of arthrogryposis are listed in Table 1.

**TABLE 1:** Clinical characteristics and outcome of 22 fetuses diagnosed with FADS

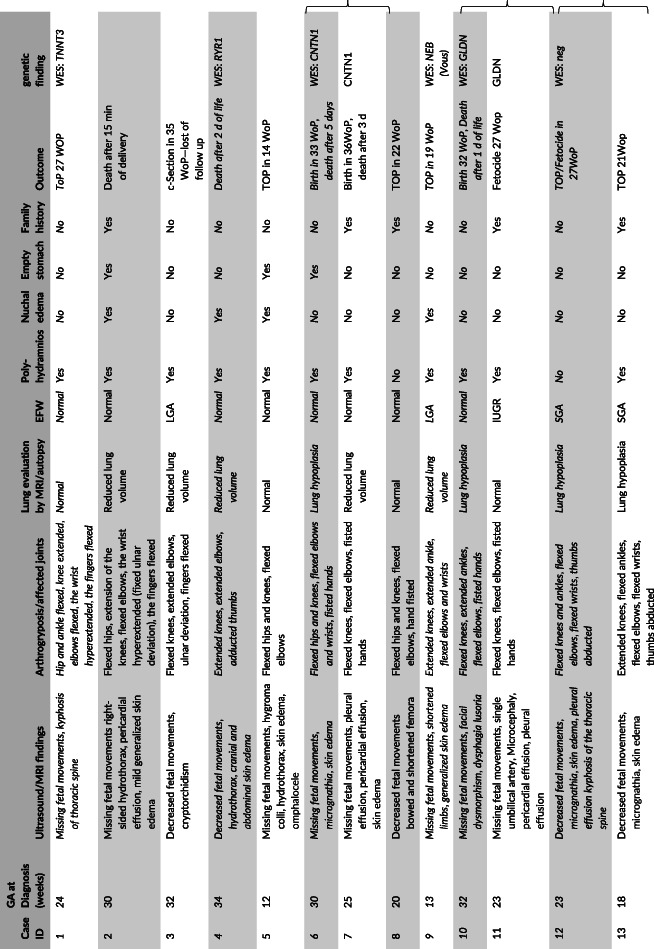 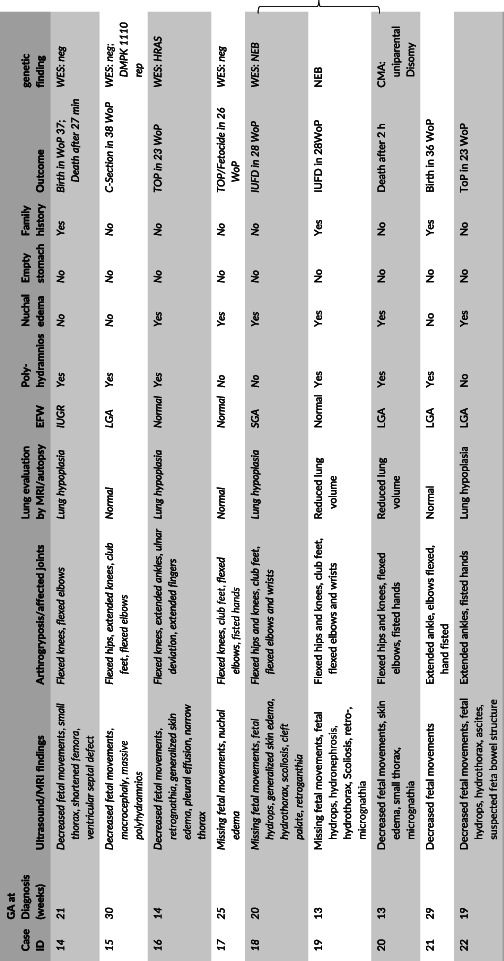

*Note*: The curly brackets mark the pair of siblings; cases with exome sequencing are marked italics.

Abbreviations: CMA, chromosomal microarray analysis; EFW, expected fetal weight; GA, gestational age; IUFD, intrauterine fetal death; IUGR, intrauterine growth restriction; LGA, large for gestational age; SGA, small for gestational age; ToP, termination of pregnancy; WoP, weeks of pregnancy.

### 4.2 Pregnancy outcome

In 10/22 (45%) cases the diagnosis led to the decision to termination of pregnancy (ToP) and in 12 cases (55%) the parents decided to continue pregnancy. ToP was performed between 14 and 27 WoP, whereas later in pregnancy, from week 24 on additional feticide was performed, which concerned four of the 10 cases. Intrauterine death occurred in two cases of the same family. Neonatal death occurred in eight of 10 live born cases (80%), mainly due to respiratory insufficiency.

## 5 GENETIC FINDINGS

### 5.1 Chromosomal microarray analysis

Chromosomal microarray analysis (CMA) was performed in all 22 euploid cases. CMA revealed a normal result in 21 cases. In case 20 a uniparental Disomy (UPD) 14 was found. In one case out of 22 cases, respectively 5% the CMA revealed a pathological result leading to a genetic diagnosis.

### 5.2 Whole exome sequencing

Exome sequencing of the index case of 11 families, revealed disease causing variants in six cases (55%) of *RYR1*, *GLDN*, *CNTN*1, *NEB*, *TNNT3* and *HRAS*, respectively.

WES revealed two compound heterozygous variants of *RYR1* in case 4: one frameshift variant leading to a premature stop codon (c.5618delA; p.Glu1873GlyfsTer57) and a missense variant (c.10018G>A, p.Val3340Met) predicted to be pathogenic. None of the two variants have been described in previous cases. The homozygous missense variant of *GLDN*, detected in the two fetuses (case 10 and 11) of a consanguineous family (c.1423G>C; p.Ala475Pro), was previously described in a case of FADS by Maluenda et al.^15^ In case 6 an intragenic deletion of the exons 2 to 15 and 18 to 19 of *CNTN1* was detected by exome sequencing, leading to the diagnosis of congenital myopathy Compton‐North. The homozygous frameshift variant of *NEB* (c.8867delA; p.Asp2956IsofsTer6), present in case 18, causing a premature stop codon, was not described in the literature before.

In case 1 a previously described heterozygous variant of *TNNT3* was detected.

Additionally, a likely pathogenic variant of *HRAS*, causing Costello syndrome, was found in case 16. All pathogenic variants detected by exome sequencing are listed in Table 2.

**TABLE 2:** Pathogenic variants identified using whole exome sequencing

Case ID	Gene symbol	Mode of inheritance	Zygosity	Variant		Relevance of variant	Phenotype described before
1	*TNNT3*	a.d.	Heterozygous	NM_006757.3: c.188G > A	NP_006748.1: p.Arg63His	Known pathogenic variant	Distal arthrogryposis type 2B reported by Sung et al^16^
4	*RYR1*	a.r.	Compound heterozygous	NM_00540: c.5618delA	NP_000531.2: p.Glu1873GlyfsTer57	Novel variant, prediction: pathogenic	
				NM_00540: c.10018G > A	NP_000531.2: p.Val3340Met	Novel variant, prediction: pathogenic	
6	*CNTN1*	a.r.	Homozygous	Deletion of exon 2‐15 and 18‐19		Novel variant, prediction: pathogenic	
10	*GLDN*	a.r.	Homozygous	NM_181789.3: c.1423G > C	NP_861454.2: p.Ala475Pro	Known pathogenic variant	Lethal FADS with lung hypoplasia reported by Maluenda et al^15^
16	*HRAS*	a.d.	Heterozygous	NM_005343.2: c.183G > C	NP_005334.1:p.Gln61His	Reported in neoplasm, classified: likely pathogenic	
18	*NEB*	a.r.	Homozygous	NM_001164508: c.8867delA	NP_001157980: p.Asp2956IsofsTer6	Novel variant, prediction: pathogenic	

Abbreviations: a.d., autosomal dominant; a.r., autosomal recessive.

As case 6 and case 7 were siblings of a consanguineous couple, we screened the other fetus for the detected homozygous variant of *CNTN1* and confirmed the diagnosis in case 7. The variant of *GLDN*, detected in case 10, was present in the second fetus of the same family case 11, confirmed by Sanger sequencing. In another consanguineous family, the homozygous variant of *NEB* (case 18) was confirmed in the sibling, case 19.

For the disease causing variants in these fetuses detected by exome sequencing the mode of inheritance was autosomal dominant in two families (33%) and autosomal recessive in four families (67%). In case 9 exome sequencing revealed two heterozygous variants (c.3986A>C/C.13328A>G) of *NEB*. Both missense variants revealed different results following two different prediction tools, so that these variants were classified as variants of unknown significance (VOUS).

Summarized, exome sequencing helped to establish a diagnosis in six of 11 families, respectively in nine of 15 cases, revealing a diagnostic yield of 55% (respectively 60%).

### 5.3 Further follow up

In case 15 exome sequencing was negative. After evaluation of the postnatal clinical course myotonic dystrophy type 1 (DM 1) was suspected and the single gene sequencing analysis of *DMPK* revealed a repeat expansion of 1110 CTG repeats, confirming the diagnosis of DM 1. In two other cases (case 3, case 21), recently found to be related to each other, the diagnosis of DM 1 was established postnatally.

## 6 DISCUSSION

In this retrospective study, we described the clinical course, ultrasound findings and genetic investigations of 22 affected fetuses. Generally, structural malformation and ultrasound findings described in our cohort are comparable with previous published papers.^7^, ^17^ In all cases reduced or missing fetal movements and as a consequence arthrogryposis were recorded. Polyhydramnios, the most common associated finding in these cases, occurred more frequently in this cohort compared to others. This is probably explained by the rather small rate of termination of pregnancy in this cohort. Associated IUGR has been reported before, but in varying frequencies from 20% to 40%.^7^, ^17^ In our collective, only 9% of fetuses showed IUGR. However, we could also show an association of fetal akinesia and large for gestational age in 27% of fetuses, which may be explained by the high rate of hydropic fetuses (37%) in our cohort.

The various time of diagnosis between 12 and 34 WoP, reported in previous investigations as well, is on one side explained by the different time of onset due to the cause of akinesia, on the other hand by the different time of referral to our department and if there was a previous affected pregnancy. As reported before, the first hint of fetal akinesia may be an elevated nuchal translucency.^17^, ^18^ All four fetuses with a clinical diagnosis before 14 WoP had a significant elevated nuchal translucency between 4.6 and 12.9 mm. Even though an abnormal nuchal translucency is an unspecific marker for various complex fetal malformations, elevated nuchal translucency should be followed up regarding decreased fetal movement to establish an early diagnosis, especially in families with positive family history.

Fetuses, diagnosed later in pregnancy (from 20 WoP onwards) mostly showed polyhydramnios as a first sign of decreased or absent fetal movements, before contractures may have been recognized.

Overall, clinical results may be slightly influenced by the comparison of continued and early‐terminated pregnancies, because some signs like IUGR, polyhydramnios, lung hypoplasia or other malformations would have developed later.

The rate of termination of pregnancy with 45% in this cohort is low compared to the investigation of Hoellen et al with 100% and Hellmund et al with 86%.^7^, ^17^ This might be explained by the late diagnosis during pregnancy. In all cases diagnosed after 26 WoP the parents decided to continue the pregnancy. Moreover, especially consanguineous families often stated religious conviction against ToP. In the remaining cases, the poor outcome with the high perinatal mortality, represents the severe phenotype and the precise clinical diagnosis performed by ultrasounds and fetal MRI.

Copy number variations (CNV) detected by Chromosomal microarray analyses in fetal akinesia seemed to be less promising compared to exome sequencing, according to our results. This is supported by the fact that the majority of literature in this field is focusing on molecular variants, rather than copy number variations. The small number of investigations using aCGH in the diagnostics of fetal akinesia syndrome focused on CNV in certain known disease associated genes like *NEB*.^19^ The case of paternal UPD 14 (case 20), primary presented with polyhydramnios, decreased fetal movements and skin edema, so that fetal akinesia was suspected.

A well‐known cause of fetal akinesia is severe congenital myotonic dystrophy 1 (DM1). Prenatally affected fetuses could present as asymptomatic ranging to severe fetal akinesia like in case 15. The majority of prenatally described DM1 cases in the literature, presented with severe polyhydramnios and club feet, comparable to case 15 of this cohort.^20^, ^21^ However, without positive family history of DM1, it is not possible to distinguish between DM1 and other disease entities of FADS.

Concerning further genetic diagnosis, we could identify the genetic cause of fetal akinesia syndrome by using exome sequencing in 6 of 11 families (55%). All of the disease‐causing genes detected in our cohort are known genes reported in FADS or related phenotypes.

Regarding *RYR1* associated disorders, symptoms are ranging from prenatally diagnosed akinesia, like in case 4, to adult‐onset muscle weakness. Similarly, patients affected by *NEB* variants show a wide disease spectrum. The severe variant of *NEB* causing a premature stop codon in case 18 and 19 may explain the severe phenotype with intrauterine fetal death.

In contrast to this *GLDN*‐associated lethal congenital arthrogryposis and congenital myopathy Compton‐North, resulting from homozygous *CNTN1* variants, are only associated with a severe phenotype, leading to death early in life, mostly after the first days, almost independent of the type of variant.^15^, ^22^

Heterozygous *TNNT3* gene variants are known to cause distal arthrogryposis type 2B. Whereas the majority of described cases with *TNNT3* gene variants are children or adults with or without congenital distal arthrogryposis,^23^ there has not been described any case in literature with absent fetal movements leading to the clinical diagnosis of fetal akinesia. Moreover, the same *TNNT3* variant has been described in a family with distal arthrogryposis, leading to a far milder phenotype.^16^

Interestingly, there was also one case (case 16) with a heterozygous *HRAS* variant detected by exome sequencing. Heterozygous variants of *HRAS* cause Castello syndrome, characterized by coarse facial features, short stature, heart defects, hypotonia and intellectual disability. Although prenatal manifestations of Costello syndrome like polyhydramnios, fetal hydrops and arthrogryposis match with the presentation of case 16, a targeted genetic diagnosis without exome sequencing would have been rather unfeasible, due to this rather unspecific features.^24^, ^25^

Comparing cases with positive WES to WES negative cases, reduced lung volume/lung hypoplasia was present more often in WES positive cases. In addition, polyhydramnios and elevated nuchal translucency was recorded more often in WES positive than WES negative cases. Potentially, presence of these ultrasound findings increase the chance of a positive WES result, but due to the small sample size it is not possible to draw a definite conclusion.

Of all limbs only cases without genetic diagnosis (case 17 and case 21) in case 17, a exome negative case, without family history, post mortem MRI showed fatty replacement of the muscle, typical for Amyoplasia.

Concerning the application of exome sequencing in a prenatal setting the diagnostic yields ranges from 10% to 57% by investigating fetuses with different sonographic abnormalities.^26^, ^27^, ^28^, ^29^, ^30^, ^31^ In this wide range of diagnostic yield, the high diagnostic rate in our investigation is most likely explained by the indication and the previous probability of an underlying genetic cause within the cohort. In particular, the rate of family history, rate of consanguinity and the severe disease entity of the fetuses influenced the diagnostic yield.

In general, due to the heterogeneous clinical presentation, the variety of possible candidate genes, the use of WES is an efficient method for genetic diagnosis, within this indication. Because of the fast and constant identification of new associated genes, clearly demonstrated by Kiefer and Hall listing 320 genes associated with arthrogryposis 2016^32^ and 402 in the gene ontology article of 2019,^33^ WES is preferable to gene panels.

The major limitation of this study is the retrospective design, which does not allow to screen for environmental factors like for example maternal antibodies. Stored DNA was available more often in cases with positive family history. Thus, positive family history and consanguinity was overrepresented in this cohort, possibly leading to a higher diagnostic yield of WES in this study.

In conclusion, the present study demonstrates a high diagnostic yield of WES in FADS, especially in eukaryote cases with positive family history of FADS and if consanguinity is known. Still, in a large part the underlying genetic cause remained unknown, whereas precise clinical evaluation in combination with WES shows to be the most efficient tool to find the disease causing variants.

## CONFLICT OF INTEREST

All authors confirm that there is no conflict of interest to declare.

## AUTHOR CONTRIBUTIONS

Theresa Reischer: Protocol/project development, Data collection and management, Data analysis and statistics, Manuscript writing/editing. Sandra Liebmann‐Reindl: Protocol/project development, Data collection and management, Genetic analysis, Data analysis, Manuscript writing/editing. Dieter Bettelheim: Project development, Manuscript editing. Sukirthini Balendran‐Braun: Manuscript editing. Berthold Streubel: Genetic analysis, Manuscript editing.

## Supporting information


**Table S1** List of genes associated with FADS

## Data Availability

The data that support the findings of this study are available from the corresponding author upon reasonable request.
