# Supplementary material for: Genetic diagnosis and clinical evaluation of severe fetal akinesia syndrome
Source: Prenat Diagn. 2020 Sep 10;40(12):1532–9. doi: 10.1002/pd.5809 (PMC7756553; doi:10.1002/pd.5809)
Supplement: Supplementary file 1 — Table S1 List of genes associated with FADS [file PD-40-1532-s001.docx]

Table S1: List of genes associated with FADS

| Gene symbol | Mode of inheritance | Disease | MIM number |
| --- | --- | --- | --- |
| *ACTA1* | autosomal dominant | Nemaline Myopathy 3 | #161800 |
| *ADCY6* | autosomal recessive | Lethal congenital contracture syndrome | #616287 |
| *BICD2* | autosomal dominant | Spinal muscular atrophy, lower extremity-predominant,2B,prenatal onset | #618291 |
| *CHRNA1* | autosomal recessive | Multiple pterygium syndrome, lethal type | #253290 |
| *CHRNB1* | autosomal recessive | Congenital myasthenic syndrome, 2C | #616314 |
| *CHRND* | autosomal recessive | Congenital myasthenic syndrome, 3B | #616322 |
| *CHRNG* | autosomal recessive | Escobar syndrome | #265000 |
| *CNTN1* | autosomal recessive | Congenital myopathy, Compton-north | #612540 |
| *CNTNAP1* | autosomal recessive | Lethal congenital contracture syndrome 7 | #616286 |
| *DNM2* | autosomal recessive | Centronuclear myopathy 1 | #160150 |
| *DOK7* | autosomal recessive | Fetal akinesia deformation sequence 3 | #618389 |
| *DPAGT1* | autosomal recessive | Congenital disorder of glycosylation | #191350 |
| *DYNC2H1* | autosomal recessive | Short-rib thoracic dysplasia 3 | #603297 |
| *ECEL1* | autosomal recessive | Distal arthrogryposis type 5D | #615065 |
| *ERBB3* | autosomal recessive | Lethal congenital contracture syndrome 2 | #607598 |
| *FLVCR2* | autosomal recessive | Proliferative vasculopathy and hydranencephaly-hydrocephaly syndrome | #225790 |
| *GBE1* | autosomal recessive | Glycogen storage disease 4 | #232500 |
| *GLDN* | autosomal recessive | Lethal congenital contracture syndrome 11 | #617194 |
| *GLE1* | autosomal recessive | Lethal congenital contracture syndrome 1 | #253310 |
| *GPR126* | autosomal recessive | Lethal congenital contracture syndrome 9 | #616503 |
| *KIF5C* | autosomal dominant | Complex cortical dysplasia with other brain malformations 2 | #615282 |
| *KLHL40* | autosomal recessive | Nemaline myopathy 8 | #615348 |
| *KLHL41* | autosomal recessive | Nemaline myopathy 9 | #615731 |
| *LMNA* | autosomal dominant | Congenital muscular dystrophy | #613205 |
| *MEGF10* | autosomal recessive | Myopathy, areflexia, respiratory distress and dysphagia, early onset | #614399 |
| *MTM1* | X-chromosomal recessive | X-linked centronuclear myopathy | #310400 |
| *MUSK* | autosomal recessive | Fetal akinesia deformation sequence 1 | #208150 |
| *MYH3* | autosomal dominant | Distal arthrogryposis 2A | #193700 |
| *MYOD1* | autosomal recessive | Lethal fetal akinesia syndrome | #159970 |
| *NEB* | autosomal recessive | Nemaline myopathy 2 | #256030 |
| *NEK9* | autosomal recessive | Lethal congenital contracture syndrome 10 | #617022 |
| *PHGDH* | autosomal recessive | Neu-laxova syndrome 1 | #256520 |
| *PIP5K1C* | autosomal recessive | Lethal congenital contracture syndrome 3 | #611369 |
| *POLG* | autosomal recessive | Mitochondrial DNA depletion syndrome 4A | #203700 |
| *RAPSN* | autosomal recessive | Fetal akinesia deformations sequence 2 | #618388 |
| *RYR1* | autosomal recessive | Minicore myopathy | #255320 |
| *SPEG* | autosomal recessive | Centronuclear myopathy 5 | #615959 |
| *SYNE1* | autosomal dominant, autosomal recessive | Emery-Dreifuss muscular dystrophy 4 | #612998 |
| *TRPV4* | autosomal dominant | Metatropic dysplasia | #156530 |
| *TUBB2B* | autosomal dominant | Complex cortical dysplasia with other brain malformations 7 | #610031 |
| *UBE1* | X-chromosomal recessive | X-linked spinal muscular atrophy | #301830 |
| *ZC4H2* | X-chromosomal recessive | Wieacker-Wolff Syndrome | #314580 |
| *ZMPSTE24* | autosomal recessive | Lethal restrictive dermopathy | #275210 |
